# Supplementary material for: An abundant quiescent stem cell population in Drosophila Malpighian tubules protects principal cells from kidney stones
Source: eLife. 2020 Mar 16;9:e54096. doi: 10.7554/eLife.54096 (PMC7093152; doi:10.7554/eLife.54096)
Supplement: Supplementary file 1. [file elife-54096-supp1.docx]

| **Key Resources Table** | | | | |
| --- | --- | --- | --- | --- |
| **Reagent type (species) or resource** | **Designation** | **Source or reference** | **Identifiers** | **Additional information** |
| genetic reagent (D. melanogaster) | *ry^506^* | Bloomington Drosophila Stock Center  (BDSC) | RRID:BDSC_4405 |  |
| genetic reagent (*D. melanogaster*) | *Oregon-R* | BDSC | BDSC:25211; RRID:BDSC_25211 |  |
| genetic reagent (*D. melanogaster*) | *N^55e11^* | BDSC | RRID:BDSC_28813 |  |
| genetic reagent (D. melanogaster) | *NRE-GFP* | BDSC | RRID:BDSC_30727 |  |
| genetic reagent (D. melanogaster) | *UAS-NICD* | BDSC | RRID:BDSC_52008 |  |
| genetic reagent (D. melanogaster) | *10Xstat-GFP* | BDSC | RRID:BDSC_26198 |  |
| genetic reagent (D. melanogaster) | *tsh-lacZ* | BDSC | RRID:BDSC_11370 |  |
| genetic reagent (D. melanogaster) | *Alp4-lacZ* | BDSC | RRID:BDSC_12285 |  |
| genetic reagent (D. melanogaster) | *Diap1-lacZ* | BDSC | RRID:BDSC_12093 |  |
| genetic reagent (D. melanogaster) | *ex-lacZ* | BDSC | RRID:BDSC_44248 |  |
| genetic reagent (D. melanogaster) | *UAS-myrRFP* | BDSC | RRID:BDSC_32222 |  |
| genetic reagent (D. melanogaster) | *UAS-ct* | BDSC | RRID:BDSC_36496 |  |
| genetic reagent (D. melanogaster) | *ct-RNAi* | BDSC | RRID:BDSC_29625 |  |
| genetic reagent (D. melanogaster) | *UAS-rpr,hid* | (Zhou et al., 1997) |  |  |
| genetic reagent (D. melanogaster) | *N-RNAi* | BDSC | RRID:BDSC_35640 |  |
| genetic reagent (D. melanogaster) | *UAS-Rac1.N17* | BDSC | RRID:BDSC_6292 |  |
| genetic reagent (D. melanogaster) | *FRT^19A^* | BDSC | RRID:BDSC_1709 |  |
| genetic reagent (D. melanogaster) | *FRT^42D^* | BDSC | RRID:BDSC_1802 |  |
| genetic reagent (D. melanogaster) | *Act5C>stop>lacZ* | BDSC | RRID:BDSC_6355 |  |
| genetic reagent (D. melanogaster) | *UAS-FLP* | BDSC | RRID:BDSC_4539 |  |
| genetic reagent (D. melanogaster) | *Puc^E69^-Gal4* | BDSC | RRID:BDSC_6762 |  |
| genetic reagent (D. melanogaster) | *c507-Gal4* | BDSC | RRID:BDSC_30840 |  |
| genetic reagent (D. melanogaster) | *Uro-Gal4* | BDSC | RRID:BDSC_44416 |  |
| genetic reagent (D. melanogaster) | *tsh-Gal4* | BDSC | RRID:BDSC_3040 |  |
| genetic reagent (D. melanogaster) | *esg-Gal4* | (Micchelli and Perrimon, 2006) |  |  |
| genetic reagent (D. melanogaster) | *cad-Gal4* | BDSC | RRID:BDSC_3042 |  |
| genetic reagent (D. melanogaster) | *tub-Gal80^ts^* | BDSC | RRID:BDSC_7018 |  |
| genetic reagent (D. melanogaster) | *tub-Gal80^ts^* | BDSC | RRID:BDSC_7019 |  |
| genetic reagent (D. melanogaster) | *Df31-GFP* | (Buszczak et al., 2007) |  |  |
| genetic reagent (D. melanogaster) | *Pvr-GFP* | Vienna Drosophila Resource Center  (VDRC) | RRID:VDRC_318162 |  |
| genetic reagent (D. melanogaster) | *UAS-upd1* | J. Urban (X. Chen lab, JHU) |  |  |
| genetic reagent (D. melanogaster) | *UAS-rCD2.RFP,UAS-GFPi, FRT40A* | BDSC | RRID:BDSC_56184 |  |
| genetic reagent (D. melanogaster) | *UAS-rCD8.GFP,UAS-rCD2i,FRT40A* | BDSC | RRID:BDSC_56185 |  |
| genetic reagent (D. melanogaster) | *42D-MARCM* | (Fox and Spradling, 2009) |  | UAS-cd8GFP hsFlp; FRT*^42D^ tub-Gal80; tub-Gal4/TM6B, Tb* |
| antibody | anti-Cut (mouse monoclonal) | Developemental Studies Hybridoma Bank (DSHB) | Cat#2B10; RRID: [AB_528186](http://antibodyregistry.org/AB_528186) | IF(1:20) |
| antibody | anti-Dl (mouse monoclonal) | DSHB | Cat#C594.9B; RRID: [AB_528194](http://antibodyregistry.org/AB_528186) | IF(1:10) |
| antibody | anti-NECD (mouse monoclonal) | DSHB | Cat#C458.2H; RRID: [AB_528408](http://antibodyregistry.org/AB_528186) | IF(1:10) |
| antibody | anti-β-Gal (mouse monoclonal) | Promega | Cat#Z3781; AB_430877 | IF(1:1000) |
| antibody | anti-β-Gal (rabbit polyclonal) | Cappel | Cat#55976; AB_2313707 | IF(1:2000) |
| antibody | anti-GFP (rabbit polyclonal) | ThermoFisher  Scientific | Cat# A-11122; RRID: AB_221569 | IF(1:2000) |
| antibody | anti-PH3 (mouse polyclonal) | Cell Signaling  Technology | Cat# 9706, RRID:AB_331748 | IF(1:500) |
| antibody | anti-RFP (rabit polyclonal) | Rockland | Cat# 600401379, RRID: AB_2209751 | IF(1:2000) |
| antibody | anti-dpErK (mouse polyclonal) | Sigma-Aldrich | Cat# M9692, RRID:AB_260729 | IF(1:500) |
| antibody | Alexa 488 goat anti-Mouse | ThermoFisher  Scientific | Cat#: A11001; RRID: AB_2534069 | IF(1:300) |
| antibody | Alexa 568 goat anti-Mouse | ThermoFisher  Scientific | Cat#: A11004; RRID: AB_2534072 | IF(1:300) |
| antibody | Alexa 488 goat anti-Rabbit | ThermoFisher  Scientific | Cat#: A11034; RRID: AB_2576271 | IF(1:300) |
| antibody | Alexa 568 goat anti-Rabbit | ThermoFisher  Scientific | Cat#: A11041; RRID: AB_2534098 | IF(1:300) |
| commercial assay or kit | Click-iT™ EdU Cell Proliferation Kit for Imaging, Alexa Fluor™ 488 dye | ThermoFisher | Cat#C10337 |  |
| commercial assay or kit | TruSeq RNA Library Prep Kit v2 | illumina | RS-122-2001 |  |
| chemical compound, drug | Allopurinol | Sigma Aldrich | Cat#A8003 |  |
| software, algorithm | IMARIS v9.2.1 | Bitplane | RRID:SCR_007370 |  |
| software, algorithm | Fiji | NIH | RRID:SCR_002285 |  |
| software, algorithm | DESeq2 | (Love et al., 2014) | RRID:SCR_015687 |  |
| software, algorithm | HISAT2 v2.1.0 | (Pertea et al., 2016) | RRID:SCR_015530 |  |
| software, algorithm | Seurat | (Stuart et al., 2019) | RRID:SCR_016341 |  |
| software, algorithm | Cell Ranger | 10X genomics | RRID:SCR_017344 |  |
